# Desmin and CD31 immunolabeling for detecting venous invasion of the pancreatobiliary tract cancers

**DOI:** 10.1371/journal.pone.0242571

**Published:** 2020-11-30

**Authors:** Junyoung Shin, Laura D. Wood, Ralph H. Hruban, Seung-Mo Hong

**Affiliations:** 1 Department of Pathology, Asan Medical Center, University of Ulsan College of Medicine, Seoul, Republic of Korea; 2 Department of Pathology, the Sol Goldman Pancreatic Cancer Research Center, Johns Hopkins Medical Institutions, Baltimore, MD, United States of America; 3 Department of Oncology, the Sol Goldman Pancreatic Cancer Research Center, Johns Hopkins Medical Institutions, Baltimore, MD, United States of America; Centro Nacional de Investigaciones Oncologicas, SPAIN

## Abstract

Although venous invasion (VI) is a poor prognostic factor for patients with pancreatobiliary tract cancers, its histopathologic characteristics have not been well described. We evaluated the patterns of VI and the added benefit provided by CD31, desmin, and dual CD31‒desmin immunolabeling for identification of VI. We included 120 surgically resected pancreatobiliary tract cancer cases—59 cases as a test set with known VI and 61 cases as a validation set without information of VI. VI was classified into three patterns: intraepithelial neoplasia-like (IN-like), conventional, and destructive. Hematoxylin and eosin (H&E) staining and CD31, desmin, and dual CD31‒desmin immunolabeling were performed. Foci number and patterns of VI were compared with the test and validation sets. More foci of VI were detected by single CD31 (*P* = 0.022) than H&E staining in the test set. CD31 immunolabeling detected more foci of the conventional pattern of VI, and desmin immunolabeling detected more foci of the destructive pattern (all, *P* < 0.001). Dual CD31‒desmin immunolabeling identified more foci of VI (*P* = 0.012) and specifically detected more foci of IN-like (*P* = 0.045) and destructive patterns (*P* < 0.001) than H&E staining in the validation set. However, dual CD31‒desmin immunolabeling was not helpful for detecting the conventional pattern of VI in the validation set. Patients with VI detected by dual CD31‒desmin immunolabeling had shorter disease-free survival (*P* <0.001) than those without VI. VI detected by dual CD31‒desmin immunolabeling was a worse prognostic indicator (*P =* 0.009). More foci of VI could be detected with additional single CD31 or dual CD31‒desmin immunolabeling. The precise evaluation of VI with dual CD31‒desmin immunolabeling can provide additional prognostic information for patients with surgically resected pancreatobiliary tract cancers.

## Introduction

Pancreatic ductal adenocarcinoma (PDAC) is a leading cause of cancer-related deaths in the United States and Korea [1,2]. Globally, it has been estimated that approximately 448,000 people will be diagnosed with and 441,000 will die of PDAC in 2017. Most patients are diagnosed with the advanced stage of the disease, with only 10% cases being diagnosed in the early stages [3]. Regional extension and metastasis at the time of diagnosis are frequent, with most of these patients being ineligible for surgery [4].

Several large studies have reported that venous invasion (VI) is an independent predictor of poor outcomes [5,6]. The described histologic appearance of VI can range from a conventional pattern to a pancreatic intraepithelial neoplasia (PanIN)-like pattern. The conventional pattern is characterized by floating cancer cells in the venous lumen, and the PanIN-like pattern has been described as well-oriented and lumen-forming neoplastic cells lining the full circumference of the vessel, with the vessel endothelium being completely replaced by cancer cells [6]. Although identifying foci of VI on hematoxylin and eosin (H&E) stained slides is the gold standard, this method is both labor-intensive and time-consuming to pathologists. Immunolabeling for endothelial cell markers has been reported to improve the detection of VI [7,8]. Here, we describe the histopathologic patterns and immunohistochemical features of VI in cancers of the pancreatobiliary system. We found that either single CD31 or dual CD31‒desmin immunolabeling are helpful for detecting foci of VI.

## Materials and methods

### Case selection

**Fig 1:**
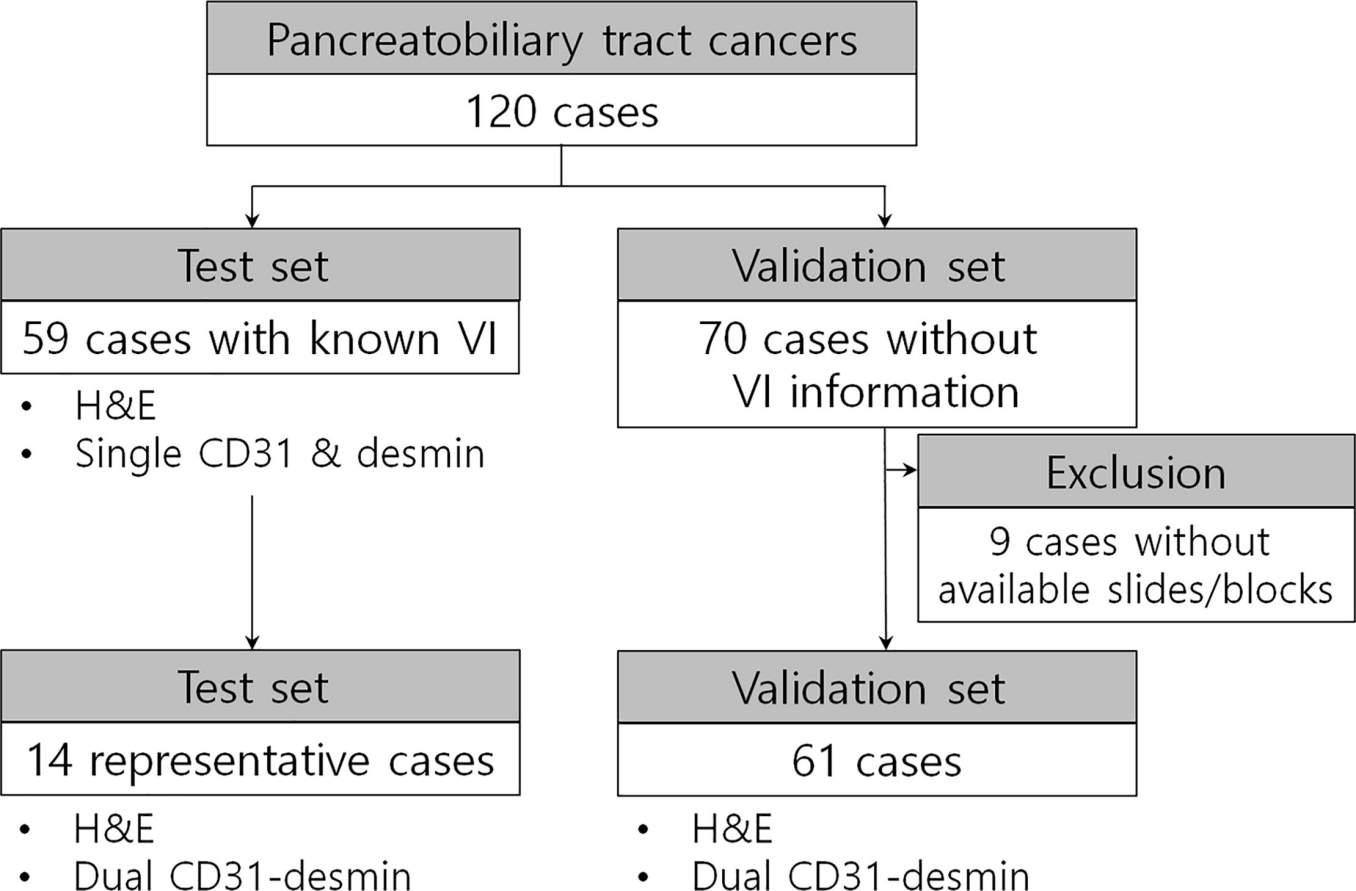
Flow diagram depicting the inclusion and exclusion criteria of the test and validation sets.

One hundred twenty-nine surgically resected pancreatobiliary tract cancer cases were included in the present study. A flow chart illustrating the inclusion and exclusion criteria is depicted in **Fig 1**. Fifty-nine cases were used as a test set with known foci of VI, which was searched from the files of the Department of Pathology. Of the 59 patients, 37 (63%) had PDAC, 19 (32%) had extrahepatic cholangiocarcinomas, and three (5%) had ampulla of Vater adenocarcinomas. An additional 70 consecutive surgically resected pancreatobiliary tract cancer cases without known information of VI status were obtained from the database of the Department of Pathology. Of them, nine cases were excluded owing to the lack of available slides or paraffin blocks. Finally, 61 cases were included in this study as the validation set.

### Ethics statement

This study was approved by the Institutional Review Board (approval number: 2020–0234) of Asan Medical Center, and the requirement for informed consent was waived. The patient’s identification codes were removed, and a new case number was randomly assigned. The electronic medical records of the patients from January 2014 to January 2018 were carefully reviewed.

### Histopathologic evaluation

VI was independently re-evaluated by two investigators, who examined all available H&E-stained slides. Because muscular veins with their branches run parallel with muscular arteries with their branches in the interlobular pancreatic connective tissues and away from the centrally located pancreatic ducts, VI was searched in areas with muscular arteries presenting without accompanying muscular vein, which was also called an orphan artery sign [9]. When a normal duct-like or PanIN-like gland was present near the muscular artery without an accompanying muscular vein, normal duct-like or PanIN-like gland was considered as VI [10]. In the test set, one representative slide containing definite foci of VI was selected, and the number of foci was counted. In addition, the histologic features of VI were classified into three patterns: conventional, intraepithelial lesion (IN)-like, or destructive. The conventional pattern was defined as incomplete coverage of the circumference of the endothelium by cancer cells [6]. The IN-like pattern, which was similar to the PanIN-like pattern of VI by pancreatic cancers, was defined as complete coverage by well-oriented cancer, forming a lumen and lining the entire circumference of the blood vessels, resulting in a neo-lumen within the blood vessels [6]. Both PanIN and IN-like patterns of the VI share neoplastic cells from the papillary architecture and have nuclear and cytologic atypia. However, the IN-like pattern of VI can be differentiated from real PanIN surrounded by a smooth muscle layer or had an orphan artery sign [6]. The term IN-like pattern was used because patients included in the present study had both pancreatic and biliary tract cancers. The destructive pattern was newly defined as destruction of the venous architecture by cancer-cell infiltration and desmoplastic fibrous stroma with a variable degree of inflammatory cell infiltration. In the validation set, the destructive pattern was further classified as suspicious or a definite destructive pattern based on the presence of residual smooth muscle layers of vein on the H&E-stained slides. When residual destructive smooth muscle layers of the venous wall were present on H&E slides, VI was categorized as the definite destructive pattern. In contrast, suspicious destructive pattern was defined when the definite residual destructive smooth muscle layers of vein were not identified, which was difficult on H&E slides; then, further single desmin or dual CD31‒desmin immunolabeling was performed to identify residual smooth muscle layers and was shown desmin positivity to confirm the destructive pattern as a definite destructive pattern.

**Fig 2:**
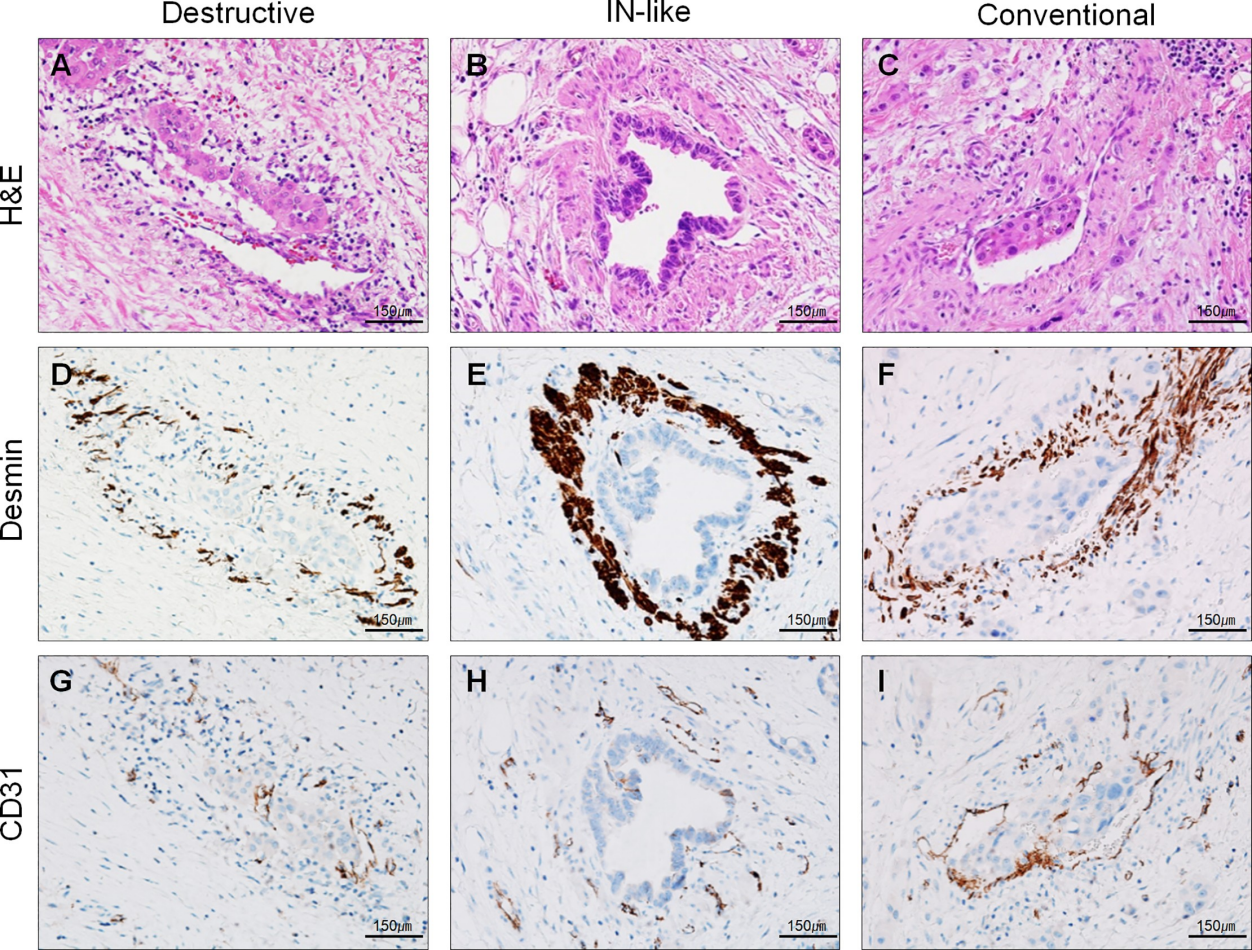
Representative H&E staining and single desmin or CD31 immunolabeling images of different patterns of venous invasion of pancreatobiliary tract cancers. Left column **(A, D, and G)**, suspicious destructive pattern. Smooth muscle layer is not identified on H&E but observed on desmin immunolabeling; central column; **(B, E, and H),** intraepithelial (IN)-like pattern, cancer cells within venous space mimic dysplastic cells of intraepithelial neoplasia showing well-circumscribe outer desmin-positive smooth muscle layer **(E)** and no CD31 expression **(H)**; right column **(C, F, and I)**, conventional pattern, well-circumscribe outer desmin-positive smooth muscle layer and cancer cells within venous space **(F)**, cancer cells within venous space attached to endothelial cells showing partial loss of CD31 expression **(I)**, while cancer cell-non-attached endothelial cells show brown color (**A-C**, H&E staining; **D-F**, desmin immunolabeling; **G-I**, CD31 immunolabeling; all ×200 magnification).

**Fig 3:**
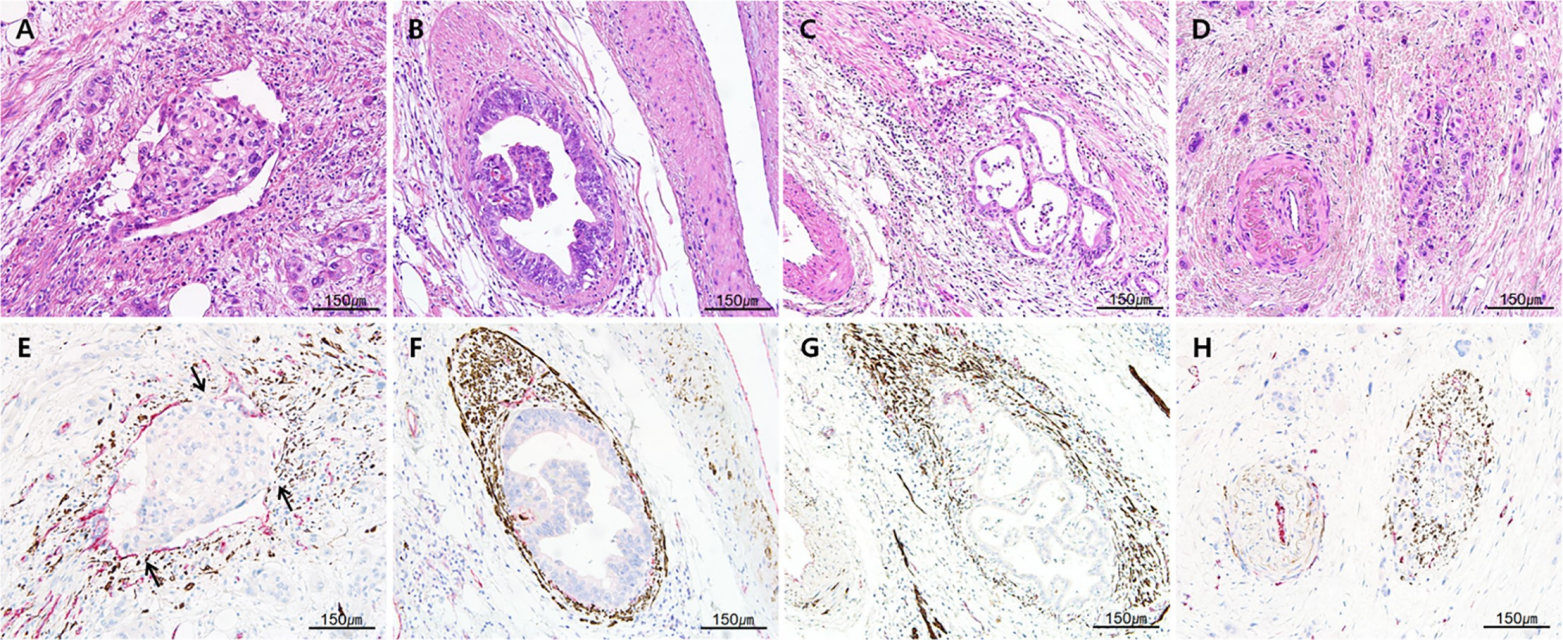
Representative H&E staining and dual CD31‒desmin immunolabeling (magenta, CD31; desmin, brown) images of different patterns of venous invasion of pancreatobiliary tract cancers. **A and E)** Conventional pattern. Cancer cells within venous space partly attach to endothelial cells showing partial loss of CD31 expression (arrows), while cancer cell-non-attached endothelial cells show magenta color (intact CD31 labeling); **B and F)** IN-like pattern; cancer cells within venous space show spotty CD31 expression (magenta color) with well- circumscribe outer desmin-positive (brown color) smooth muscle layer; **C and G)** definite destructive pattern of venous invasion on H&E staining **(C)**. Some parts of smooth muscular layer are destroyed by infiltrating cancer cells and remnant smooth muscle layer of muscular vein is identified on the H&E-stained (**C**) and confirmed by desmin (**G**; brown color) immunolabeled slide; **D and H)** suspicious destructive pattern of venous invasion on H&E staining (**D**). The single artery sign is observed; however, almost all smooth muscular layer are destroyed by infiltrating cancer cells and the equivocally remnant smooth muscle layer of muscular vein cannot be definitely identified on the H&E-stained slide (**D**) but is confirmed by the desmin (**H**; brown color) immunolabeling. (**A-D**, H&E staining; **E-H**, dual CD31‒desmin immunolabeling, CD31, magenta color; desmin, brown color; ×200 magnification).

All available H&E-stained slides of the 61 cases of the validation set were reviewed for evaluation of VI. One representative formalin–fixed and paraffin–embedded (FFPE) tissue block containing suspicious or definite foci of VI was selected after H&E-stained slide review. Dual CD31‒desmin labeling was performed on a serial sectioned unstained slide. The number of foci of VI on H&E-stained and dual CD31‒desmin labeling slides was counted independently in the H&E-stained and dual CD31‒desmin labeling slides by the same method performed for the test set. Representative images of conventional, IN-like, definite and suspicious destructive patterns of VI are depicted in **Figs 2 and 3**.

### Immunohistochemical labeling

Four μm thick tissue sections were deparaffinized and re-hydrated by immersion in xylene and a graded ethanol series. Endogenous peroxidase was blocked by incubation in 3% H_2_O_2_ for 10 minutes, followed by heat-induced antigen retrieval. Immunohistochemical labeling was performed using an autostainer (Benchmark XT; Ventana Medical Systems, Tucson, AZ, USA) according to the manufacturer’s protocol. Briefly, sections were incubated at room temperature for 24 or 32 minutes with primary antibodies against desmin (D33, 1:200; Dako, Glostrup, Denmark), and CD31 (EP78, 1:800; Cell Marque, CA, USA) and then washed. Additional AP-conjugated mouse monoclonal CD31 (EP78, 1:800; Cell Marque) was used for dual CD31‒desmin labeling. An ultraView AP Magenta Detection Kit (Ventana Medical Systems) was used for magenta chromogen, and the OptiView DAB Detection Kit (Ventana Medical Systems) was used for the brown chromogen. Immunolabeled sections were lightly counterstained with hematoxylin, dehydrated in ethanol, and cleared in xylene. The immunolabeled slides were reviewed by two pathologists. The invasion patterns of each immunohistochemical stained slide were determined. The endothelial CD31 expression was classified as “intact”, “partial (labeling of 1–359° of luminal circumference)”, and “complete” loss. Dual CD31-desmin immunolabeling was performed on 14 representative cases containing typical features of each pattern of VI on the test set, and all 61 cases on the validation set. CD31 was labeled in magenta color, and desmin in brown for dual CD31-desmin labeling. Focus-by-focus comparison was performed between H&E-stained slides and consecutive serially sectioned dual CD31-desmin immunolabeled slides.

### Statistical analysis

All statistical analyses were performed with SPSS version 19 (SPSS Inc., Chicago, IL, USA). The normality of each group was tested by the Kolmogorov–Smirnov and Shapiro–Wilk tests. The Freidman test, followed by the Wilcoxon signed rank test, was employed to compare number of detected invasion foci in the H&E, CD31, and desmin stained slides. Associations between categorical variables were examined using χ^2^ and Fisher’s exact tests. Disease-free survival rates were calculated using the Kaplan–Meier method, and statistical significances were evaluated using the log-rank test and the Cox proportional hazards regression model. A P value of less than 0.05 was considered statistically significant.

## Results

### Clinicopathologic characteristics of the patients

The clinicopathologic characteristics of the 59 patients of the test set are summarized in **Table 1**. Briefly, the mean (± standard deviation) age was 67.7 ± 7.9 years, with a male-to-female ratio of 1.3:1. The mean tumor size was 3.1 ± 1.3 cm (median, 2.8 cm; range, 1.4–9.5 cm). Of the 59 patients, 37 (63%) had been diagnosed with PDAC, 19 (32%) with extrahepatic cholangiocarcinomas, and three (5%) with ampulla of Vater adenocarcinomas. Surgery included pylorus-preserving pancreaticoduodenectomy for 38 patients (65%), distal pancreatectomy for six patients (10%), and right or left hepatectomy with bile duct resection for 15 patients (25%). Thirty-six patients (61%) exhibited lymph node metastasis, and 51 (86%) had perineural invasion. None of these patients received neoadjuvant therapy prior to surgical resection of the pancreatobiliary tumors. Fifty-one patients (86%) with recurrent disease were observed, and the median disease-free survival months were 29.5 (range, 1–99.3) months.

**Table 1:** Clinicopathologic characteristics of the test and validation sets.

	Test set	Validation set	P value
Age (mean ± SD)	67.7 ± 7.9	68.5 ± 9.9	0.076
Sex (male: female)	1.3:1	1.3:1	0.873
Tumor size (mean ± SD)	3.1 ± 1.3	3.2 ±1.8	0.100
Differentiation			0.269
Well	12 (20%)	17 (28%)	
Moderate	41 (69%)	40 (65%)	
Poor	6 (10%)	4 (7%)	
Primary tumor			0.228
Pancreas	37 (63%)	41 (67%)	
Ampulla of Vater	3 (5%)	13 (21%)	
Biliary tract	19 (32%)	7 (11%)	
Surgical procedure			0.007*
PPPD	38 (65%)	43 (70%)	
Distal pancreatectomy	6 (10%)	14 (23%)	
Hepatectomy with BDR	15 (25%)	4 (7%)	
Perineural invasion (%)	51 (86%)	42 (69%)	0.022*
Lymph node metastasis (%)	36 (61%)	33 (54%)	0.403
Recurrence	51 (86%)	40 (66%)	0.028*
Disease-Free Months (months, range)	29.5 (1–99.3)	22.3 (1–43.1)	0.065

*Statistically significant at *P* < 0.05.

Abbreviations: SD, standard deviation; PPPD, pylorus-preserving pancreaticoduodenectomy; BDR, bile duct resection.

The clinicopathologic characteristics of the 61 patients of the validation set are also summarized in **Table 1**. The mean (± standard deviation) age was 68.5 ± 9.9 years, with a male-to-female ratio of 1.3:1. The mean tumor size was 3.2 ± 1.8 (median 2.9; range 1.0–10.3 cm). Of the 61 patients of the validation set, 41 (67%) had been diagnosed with PDAC, 7 (11%) with extrahepatic cholangiocarcinomas, and 13 (21%) with ampulla of Vater adenocarcinomas. Surgery included pylorus-preserving pancreaticoduodenectomy for 43 patients (70%), distal pancreatectomy for 14 patients (23%), and right or left hepatectomy with bile duct resection for four patients (7%). Thirty-three patients (54%) exhibited lymph node metastasis, and 42 (69%) had perineural invasion. VI in 39 (64%) cases was detected by H&E-stained slides. Thirty-two patients (52%) died of the disease, and 40 patients (66%) experienced recurrence. The median disease-free survival months were 22.3 (range, 1–43.1) months. Perineural invasion was more commonly observed in the test set than in the validation set (*P* = 0.022). Surgical procedures were different between the two sets (*P* = 0.007*)*. However, no significant differences were observed in comparison between clinicopathologic factors, including age, sex, tumor size, location, and differentiation, between the test and validation sets.

### Foci of VI detected by H&E and immunohistochemical staining in the test set

The mean 13.7 ± 3.5 H&E-stained slides per case (range 7–23 slides) were reviewed. The foci numbers of VI detected by H&E staining and by CD31 and desmin immunolabeling are summarized in **Tables 2** and **3**.

**Table 2:** Foci number of VI based on invasion patterns and staining methods in the test set.

Staining	Total foci number of VI (mean ± SD/case)	Foci number (%) of VI		
		Conventional	IN-like	Destructive
H&E	181 (3.1 ± 3.1)	37 (20.4%)	131 (72.4%)	13 (7.2%)
CD31	206 (3.5 ± 3.3)	68 (33.0%)	134 (65.0%)	4 (2.0%)
Desmin	202 (3.4 ± 3.3)	29 (14.4%)	128 (63.3%)	45 (22.3%)
*P* value	0.020^*^	<0.001^*^	0.887	<0.001^*^

*Statistically significant at *P* < 0.05.

Abbreviations: VI, venous invasion; SD, Standard deviation; IN-like, intraepithelial-like; H&E, hematoxylin and eosin.

**Table 3:** Comparisons of vascular invasion patterns based on staining methods in the test set.

Staining	Invasion pattern	H&E	CD31	Desmin
H&E	Total		0.022^*^	0.027^*^
	Conventional		<0.001^*^	0.097
	IN-like		0.788	0.540
	Destructive		0.083	<0.001^*^
CD31	Total			0.911
	Conventional			<0.001^*^
	IN-like			0.644
	Destructive			<0.001^*^

*Statistically significant at *P* < 0.05.

Abbreviations: IN-like, intraepithelial-like; H&E, hematoxylin and eosin.

**Fig 4:**
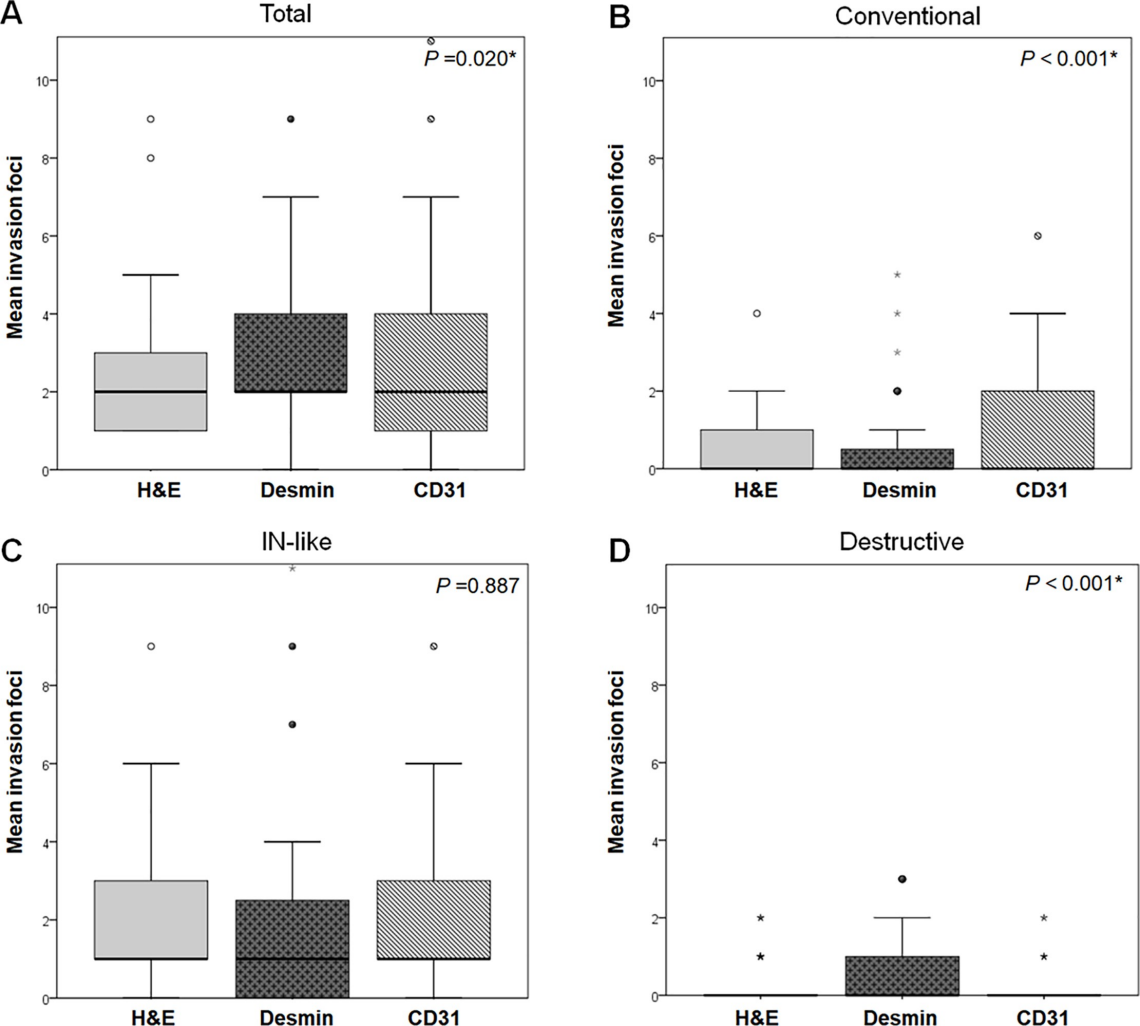
Comparisons of mean foci number of VI of pancreatobiliary tract cancers in the test set. **(A)** Total foci of venous invasion regardless of invasion pattern. Invasive foci of the **(B)** conventional, **(C)** IN-like, and **(D)** destructive patterns, as assessed by H&E staining and desmin and CD31 immunolabeling in the test set. **(A)** The mean total number of foci of VI regardless of invasion pattern detected by CD31 (*P* = 0.022) and desmin (*P* = 0.027) immunolabeling is significantly higher than that detected by H&E staining. **(B)** CD31 immunolabeling detected more invasion foci of the conventional pattern of VI than H&E staining (*P* < 0.001). **(C)** There is no significant difference in the detection of the IN-like pattern of VI by H&E staining, desmin, and CD31 immunolabeling. **(D)** Desmin immunolabeling detected more invasion foci of the destructive pattern (*P* < 0.001).

Briefly, 181, 206, and 202 foci of VI were detected by H&E staining, CD31, and desmin immunolabeling, respectively. The number of foci detected by CD31 and desmin immunolabeling was significantly higher than that detected by H&E staining (*P* = 0.020). The mean numbers of foci of VI per case detected by H&E staining and by CD31 and desmin labeling were 3.1 ± 3.1, 3.5 ± 3.3, and 3.4 ± 3.3, respectively. The Kruskal–Wallis test and Wilcoxon rank sum test were employed for pair-wise comparison because these parameters were not distributed normally. The number of invasive foci detected by CD31 (*P* = 0.022) and desmin (*P* = 0.027) immunolabeling was significantly greater than that detected by H&E staining. However, the difference between the numbers of foci of VI detected by CD31 and desmin immunolabeling was not statistically significant (*P* = 0.911; **Fig 4**).

Of the 181 foci detected by H&E staining, 131 (72.4%) foci were IN-like, 37 (20.4%) foci were conventional, and 13 (7.2%) foci were destructive pattern. Of the 206 foci detected by CD31 labeling, 134 (65.0%) showed an IN-like pattern, 68 (33.0%) showed a conventional pattern, and four (2.0%) showed a destructive pattern. Similarly, of the 202 venous invasion foci detected by desmin labeling, IN-like foci were 128 (63.3%), conventional foci were 29 (14.4%), and destructive foci were 45 (22.3%).

The total number of foci of VI detected by desmin (*P* = 0.027) and CD31 (*P* = 0.022) immunolabeling was significantly higher than that detected by H&E staining regardless of the invasion pattern of VI (**Fig 4A**). Similarly, significantly more foci of the conventional pattern of VI were detected by CD31 labeling (mean, 1.2 ± 1.5) than by H&E staining (0.6 ± 0.9; *P* < 0.001; **Fig 4B**) or desmin labeling (0.5 ± 1.0; *P* < 0.001; **Fig 4B**). Although the IN-like pattern was the most commonly detected pattern of VI by the three staining methods, pair-wise comparisons between H&E staining and CD31 labeling (*P* = 0.788), H&E staining and desmin (*P* = 0.540), and CD31 and desmin staining (*P* = 0.644), showed no significant differences between the staining methods for detecting the IN-like pattern (**Fig 4C)**. Significantly more foci of the destructive pattern of VI were detected by desmin labeling (mean number of invasion foci per case, 0.8 ± 1.0) than by H&E staining (0.2 ± 0.5; *P* < 0.001; **Fig 4D**) or CD31 labeling (0.1 ± 0.3; *P* < 0.001; **Fig 4D**).

### Mixed patterns of VI in the test set

**Fig 5:**
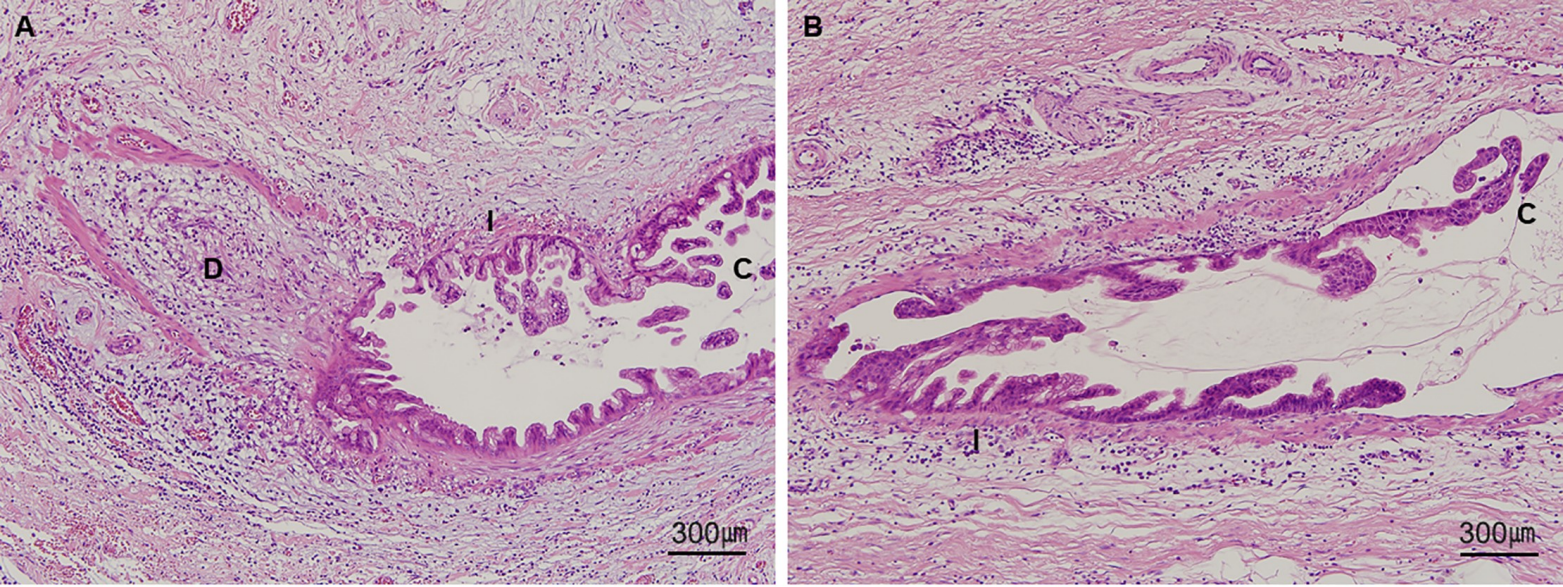
Representative images of mixed patterns of VI. **(A)** A focus of VI showing all three patterns—destructive (D), IN-like (I), and conventional (C). This rapid transition from destructive to IN-like and finally to conventional patterns suggests that these three patterns are strongly associated between each pattern. **(B)** VI exhibiting mixed patterns showing transition from IN-like (I) to conventional (C) patterns (all, ×100 magnification).

Interestingly, a few cases contained mixed patterns of VI. For example, the focus of VI in **Fig 5A** exhibited all three patterns—destructive, IN-like, and conventional. Similarly, another focus of VI in **Fig 5B** showed both IN-like and conventional patterns. This rapid transition from destructive to IN-like and finally to conventional patterns suggests that these three patterns are strongly associated.

### Qualification of the CD31 expression of VI in the test set

**Fig 6:**
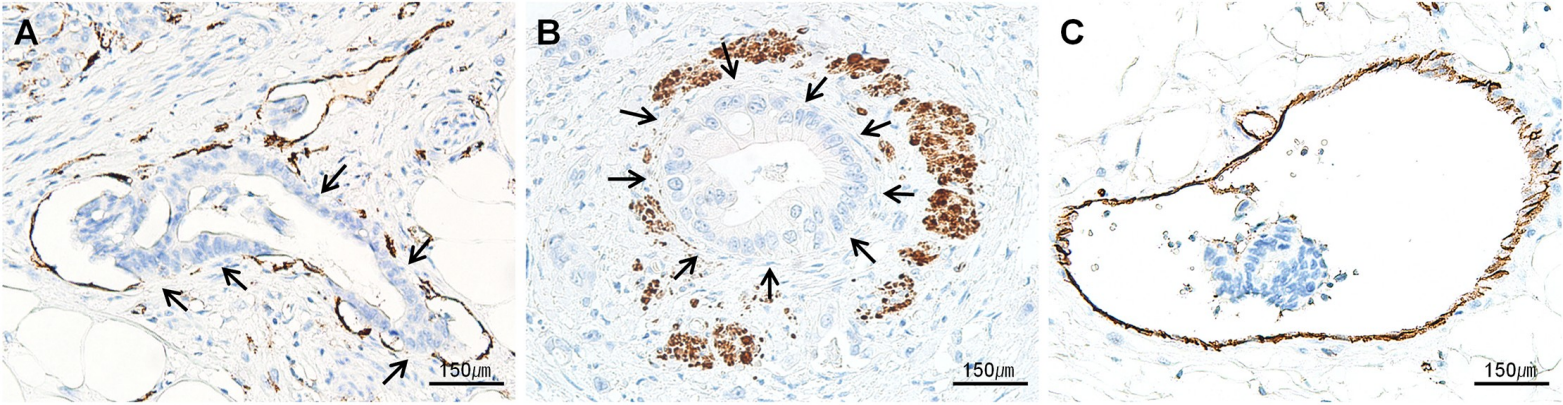
Representative images of CD31 immunolabeling of VI. **(A)** Partial loss of CD31 labeling (arrows) of endothelial cells where attachment of cancer cells on the endothelial portion. **(B)** Dual CD31 and desmin immunolabeling showing total loss of CD31 labeling on endothelial cells where attachment of cancer cells on the entire endothelial cells (arrows) with outer desmin positive smooth muscle of muscular vein. **(C)** Intact CD31 labeling of endothelial cells (all, ×400 magnification).

Invasive foci showed various degrees of loss of CD31 expression. Of the 206 foci of VI, 160 (77.7%) exhibited partial loss (**Fig 6A**) and 39 foci (18.9%) exhibited complete loss of expression (**Fig 6B**), whereas only seven foci (3.4%) showed intact expression (**Fig 6C**).

Of the 68 foci showing the conventional pattern, 60 (88.2%) showed partial loss of CD31 expression, seven (10.3%) showed intact CD31 labeling, and one (1.5%) showed a complete loss of CD31 expression. Of the 134 foci with an IN-like pattern, 96 (71.6%) showed partial loss and 38 (28.4%) showed complete loss of CD31 expression, with none showing intact CD31 labeling. By contrast, all four foci with a destructive pattern showed partial CD31 loss, and none exhibited total loss or intact CD31 expression.

### Dual CD31‒desmin immunolabeling of test set

Dual CD31‒desmin immunolabeling was performed on samples from 14 representative patients with 62 foci of VI, including 26 conventional, 32 IN-like, and 4 destructive patterns on the test set. All 62 foci were easily detected by dual CD31 and desmin labeling, regardless of the invasion patterns (**Fig 3).**

### VI Foci detected by H&E staining and dual CD31‒desmin immunolabeling of the validation set

**Fig 7:**
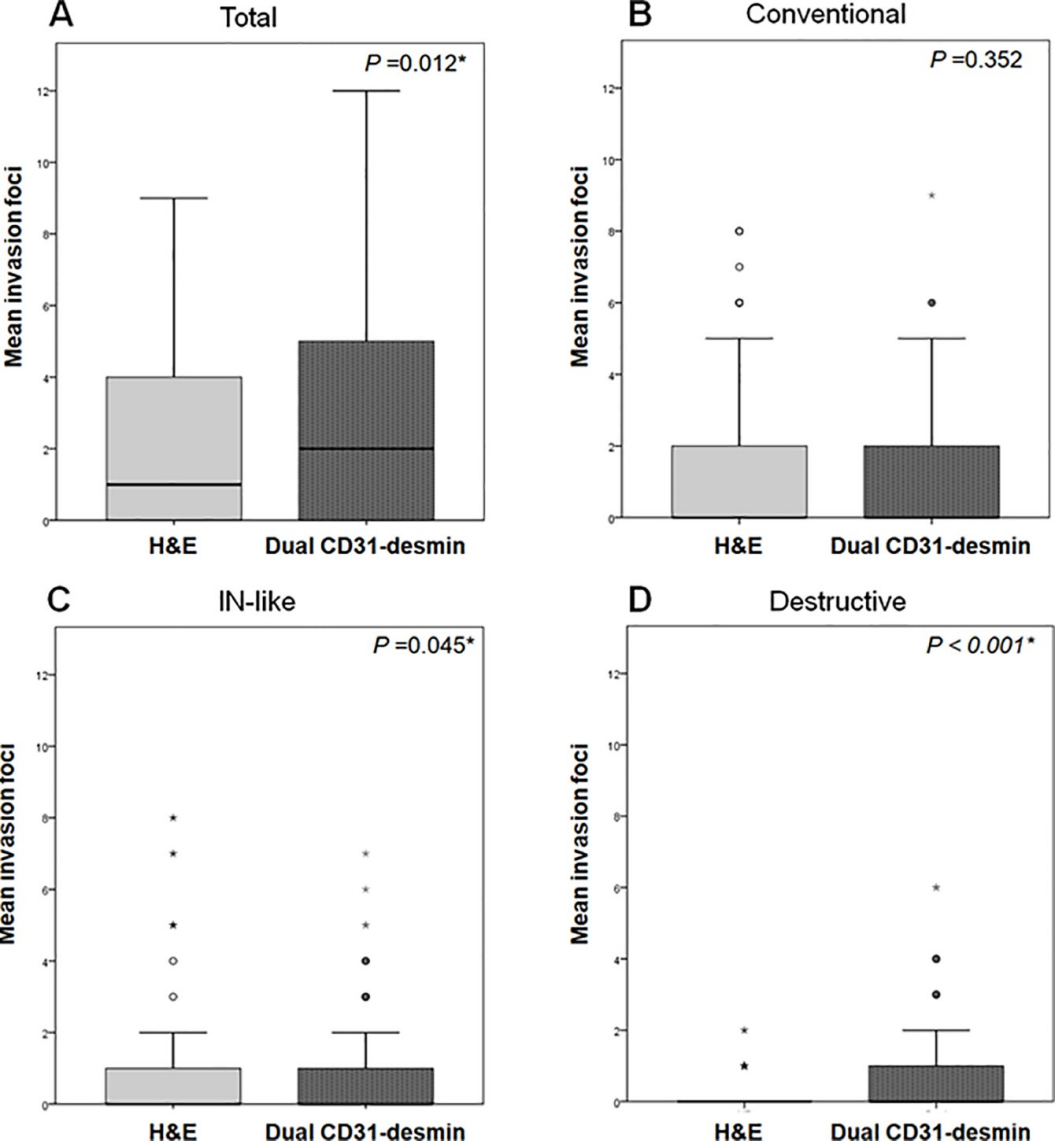
Comparisons of mean number of foci of VI of pancreatobiliary tract cancers on the validation set. **(A)** Total foci of VI regardless of invasion pattern and invasive foci exhibiting the **(B)** conventional, **(C)** IN-like, and **(D)** destructive patterns as assessed by H&E staining and dual CD31‒desmin immunolabeling among the validation set. **(A)** Dual CD31‒desmin immunolabeling detected more invasion foci of VI regardless of the invasion pattern than H&E staining (*P* = 0.012). **(B)** No significant difference was observed in the detection of the conventional pattern of VI between H&E staining and dual CD31‒desmin immunolabeling. **(C)** Dual CD31‒desmin immunolabeling detected more invasion foci exhibiting the IN-like pattern of VI than H&E staining (*P* = 0.0045). **(D)** Similarly, dual CD31‒desmin immunolabeling detected more invasion foci exhibiting the destructive pattern than H&E staining (*P* < 0.001).

The number of VI foci detected by H&E and dual CD31‒desmin staining are summarized in **Table 4**. Briefly, a total of 146 and 194 foci of VI were detected by H&E staining and dual CD31‒desmin immunolabeling. Significantly more foci of VI were detected by dual CD31-desmin immunolabeling (mean, 3.2 ± 3.0 foci) than H&E staining (2.4 ± 2.9; *P* = 0.012). Of the 146 foci of VI detected by H&E staining, 87 (59.6%) exhibited the conventional pattern, 50 (34.2%) the IN-like pattern, and nine (6.2%) the destructive pattern. Most VI detected by H&E staining exhibited either the conventional or IN-like pattern, which consisted of 93.8% of VI among the validation set. Of the 194 foci detected by dual CD31‒desmin immunolabeling, 62 (32.0%) exhibited an IN-like pattern, 74 (38.2%) the conventional pattern, and 58 (29.8%) the destructive pattern. Dual CD31‒desmin immunolabeling (mean number of invasion foci per case, 0.9 ± 1.4) detected significantly more foci with destructive pattern of VI than H&E staining (0.1 ± 0.4; *P* < 0.001; **Fig 7**). Similarly, dual CD31‒desmin immunolabeling (mean number of invasion foci per case: 1.0 ± 1.7) detected significantly more foci exhibiting the IN-like pattern of VI than H&E staining (0.8 ± 1.7; *P* = 0.045). However, no difference was observed in the detection of the conventional pattern between H&E staining and dual CD31‒desmin immunolabeling (P = 0.352).

**Table 4:** Focus-by-focus comparisons of vascular invasion patterns observed by H&E and dual CD31‒desmin immunolabeling of the validation set.

Staining Invasion pattern	H&E	Dual CD31-desmin	*P-value*
Total (mean ± SD)	146 (2.4 ± 2.9)	194 (3.2 ± 3.0)	0.012^*^
Conventional (mean ± SD)	87 (59.6%, 1.4 ± 2.2)	74 (38.2%, 1.2 ± 1.9)	0.352
IN-like (mean ± SD)	50 (34.2%, 0.8 ± 1.7)	62 (32.0%, 1.0 ± 1.7)	0.045^*^
Destructive (mean ± SD)	9 (6.2%, 0.1 ± 0.4)	58^#^ (29.8%, 0.9 ± 1.4)	<0.001^*^

*Statistically significant at *P* < 0.05.

Abbreviations: SD, Standard deviation; IN-like, intraepithelial-like; H&E, hematoxylin and eosin.

^#^58 foci exhibiting the destructive pattern after dual CD31-desmin labeling included nine definite foci with a remnant smooth muscle layer in the H&E-stained samples, 13 confirmed foci from 25 suspicious foci with equivocally remnant smooth muscle layer in the H&E-stained samples, and 36 newly detected foci in the dual labeled samples that were not identified by H&E staining.

On the H&E-stained slides, nine definite and 25 suspicious foci of the destructive pattern were observed. However, 58 foci were finally confirmed as exhibiting the destructive pattern after dual CD31-desmin labeling, which included nine definite foci among the H&E-stained samples, 13 confirmed foci from 25 suspicious foci among the H&E-stained samples, and 36 newly detected foci among the samples that were dual CD31‒desmin immunolabeled but were not identified by H&E staining.

### Association between VI and other clinicopathologic factors

Pancreatobiliary tract cancers with VI tended to have a high rate of perineural invasion (VI absent, 11 cases, 52% vs. VI present, 31 cases, 78%, *P* = 0.044) and recurrence (VI absent, 7 cases, 33% vs. VI present, 33 cases, 83%, *P* <0.001) than those without VI (**Table 5**). Tumor size was slightly larger in the VI present group; however, the results were not statistically significant (VI absent, 2.6 ± 1.6 vs. VI present, 3.5 ± 1.8, *P* = 0.053). No difference between the two groups was observed in terms of age, male-to-female ratio, differentiation, primary site, and status of lymph node metastasis.

**Table 5:** Association between VI and other clinicopathologic features in pancreatobiliary tract cancers.

Characteristics	Venous invasion		*P* value
	Absent	Present	
Number of cases	21	40	
Age (mean ± SD)	65.9 ± 10.9	69.9 ± 9.2	0.128
Sex (male: female)	1.1:1	1.5:1	0.568
Size (cm ± SD)	2.6 ± 1.6	3.5 ± 1.8	0.053
Differentiation			0.294
Well	7 (33%)	10 (25%)	
Moderately	14 (67%)	26 (65%)	
Poorly	0 (0%)	4 (10%)	
Primary tumor			0.179
Pancreas	11 (52%)	30 (75%)	
Ampulla of Vater	7 (33%)	6 (15%)	
Bile duct	3 (15%)	4 (10%)	
Perineural invasion	11 (52%)	31 (78%)	0.044*
Lymph node metastasis	9 (43%)	24 (60%)	0.202
Recurrence	7 (33%)	33 (83%)	<0.001*
Disease-free survival months (median, range)	36.4 (11.5–43.1)	27.1 (1.5–41.5)	< .0001*

*Statistically significant at *P* < 0.05.

Abbreviations: SD, Standard deviation; IN-like, intraepithelial-like; H&E, hematoxylin and eosin.

### Patient survival and VI

**Fig 8:**
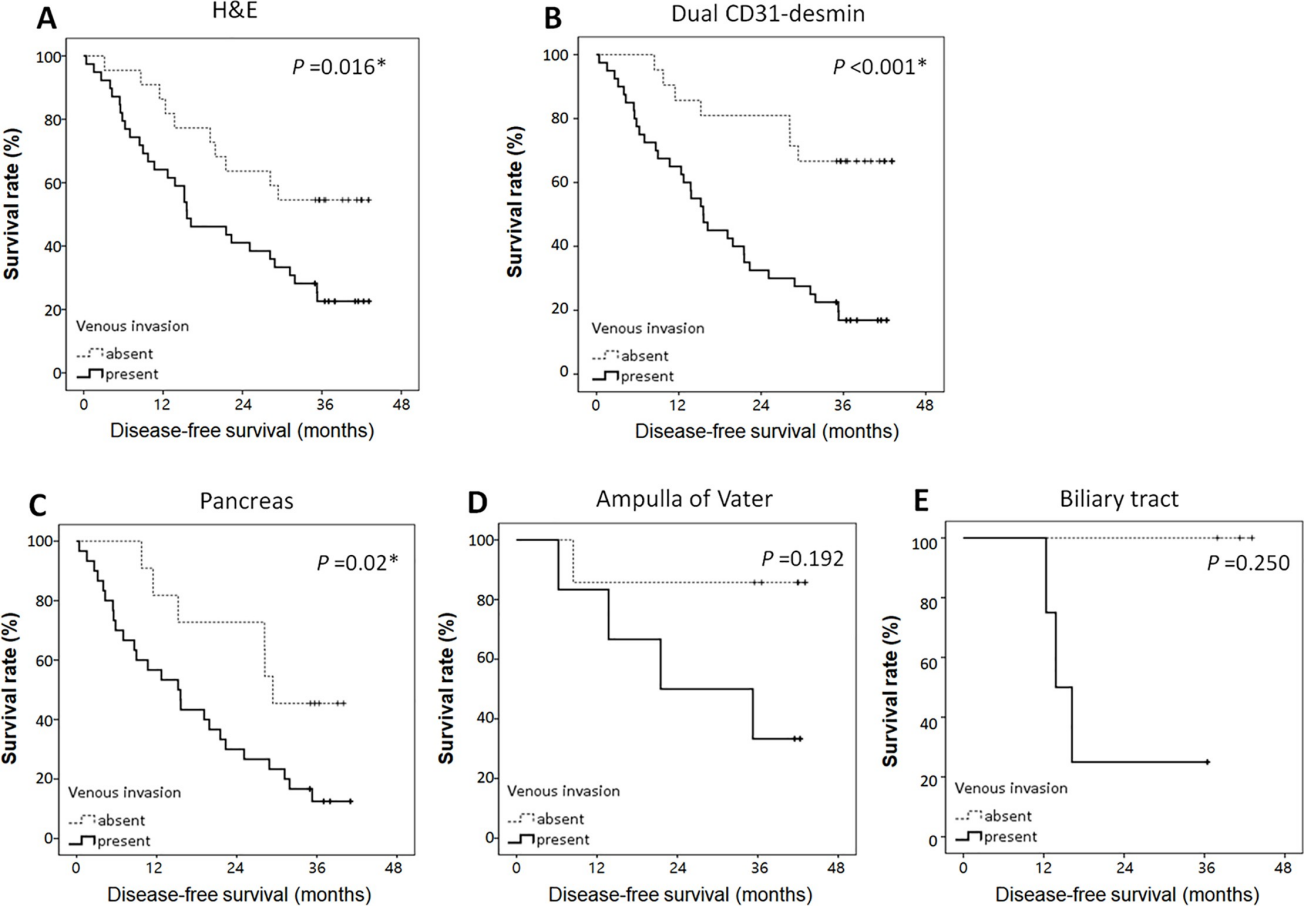
Kaplan–Meier survival analyses of disease-free survival of pancreatobiliary tract cancers based on VI status. **(A)** Patients with pancreatobiliary tract cancer and venous invasion detected by H&E staining have significantly worse disease-free survival (3-year survival rate, 23%) than those without VI (46%; *P* = 0.016). **(B)** Patients with pancreatobiliary tract cancer and venous invasion detected by dual CD31‒desmin immunolabeling have significantly worse disease-free survival (3-year survival rate, 18%) than those without venous invasion (67%; *P* <0.001). **(C)** Patients with pancreas cancer and venous invasion have significantly shorter disease-free survival (3-year survival rate, 14%) than those without venous invasion (42%; *P* = 0.02) by dual CD31‒desmin immunolabeling. Patients with **(D)** ampulla of Vater and **(E)** biliary tract cancers and venous invasion had a tendency of shorter disease-free survival than those without venous invasion (ampulla of Vater cancer, 3-year survival rate, 34% vs 86%, *P* = 0.192; biliary tract cancer, 100% vs 25%, *P* = 0.250) by dual CD31‒desmin immunolabeling.

The disease-free survival was significantly shorter for patients with pancreatobiliary tract cancers and VI detected by H&E staining (3-year survival rate, 23%) than those without VI (55%; *P* = 0.016; **Fig 8A**). Similarly, the 3-year disease-free survival was significantly shorter for patients with pancreatobiliary tract cancers and VI detected by dual CD31‒desmin immunolabeling (3-year survival rate, 18%) than those without VI (67%; *P* <0.001; **Fig 8B**). Patient survival based on VI status was also compared according to the location of primary tumors (pancreas, ampulla of Vater, and biliary tract). Patients with pancreas cancer and VI had significantly shorter disease-free survival (3-year survival rate, 14%) than those without VI (42%; *P* = 0.02; **Fig 8C**). However, patients with ampulla of Vater (3-year survival rate, 34% vs 86%; *P* = 0.192; **Fig 8D**) and biliary tract cancers (3-year survival rate, 100% vs 25%; *P* = 0.250; **Fig 8E**) with VI showed a tendency of shorter disease-free survival by dual CD31‒desmin immunolabeling.

### Univariate analysis for other clinicopathologic factors

The median length of follow-up among all 61 patients in the validation set was 35.5 (range, 1 to 43.6) months, and the median disease-free survival for all 61 patients in the validation set was 22.3 (range, 1 to 43.1) months. In univariate survival analysis, the following clinicopathologic factors were associated with shorter disease-free survival (**Table 6**), patient age (*P* = 0.006), poor tumor differentiation (*P* < 0.001), perineural invasion (*P* = 0.002), and lymph node metastasis (*P* = 0.003).

**Table 6:** Univariate and multivariate analyses of pancreatobiliary tract cancer in the validation set.

	Univariate analysis			Multivariate analysis (H&E VI)		Multivariate analysis (dual VI)	
Characteristics	Total	OR (95% CI)	*P* value	OR (95% CI)	*P* value	OR (95% CI)	*P* value
Age, years		1.05 (1.0–1.1)*	0.006*	1.03 (0.99–1.1)	0.129	1.05 (1.0–1.1)	0.06
Sex							
Male	35	1					
Female	26	1.59 (0.8–3.0)	0.150				
Size, cm		1.11 (0.95–1.3)	0.181				
Location							
Pancreas	41	1					
Ampulla of Vater	13	0.77 (0.2–1.4)	0.128				
Biliary tract	7	0.84 (0.4–2.1)	0.813				
Differentiation							
WD	17	1					
MD	40	1.95 (0.89–4.3)	0.096	0.83 (0.3–1.9)	0.686	1.15 (0.5–2.9)	0.761
PD	4	14.41 (3.5–58.9)*	<0.001*	11.09 (2.7–45.2)*	0.001*	11.15 (2.9–42.4)*	<0.001*
PNI							
Absent	19	1					
Present	42	3.95 (1.6–9.5)*	0.002*	**3.37 (1.2–9.5)**	0.021*	3.41 (1.2–9.5)*	0.019*
LN metastasis							
Absent	28	1					
Present	33	2.81 (1.4–5.5)*	0.003*	**2.10 (1.0–4.5)**	0.038*	1.70 (0.8–3.6)	0.163
VI detected by H&E staining							
Absent	22	1					
Present	39	2.31 (1.1–4.7)*	**0.022**	1.96 (0.9–4.6)	0.088		
VI detected by dual CD31‒desmin labeling							
Absent	21	1					
Present	40	4.12 (1.8–9.4)*	0.001*			3.31 (1.3–7.3)*	0.009*

*Statistically significant at *P* < 0.05.

Abbreviations: WD, well differentiated; MD, moderately differentiated; PD, poorly differentiated; PNI, perineural invasion; H%E VI, vascular invasion evaluated by H&E staining; Dual VI, vascular invasion evaluated by dual CD31‒desmin immunolabeling.

### Multivariate analyses

Multivariate analyses were performed to determine which factors remained independent predictors of disease-free survival after adjustment for factors that were significant in univariate analyses. The analyses were conducted by alternately including the VI status evaluated by H&E staining and dual CD31‒desmin immunolabeling (**Table 6**).

Multivariate analysis showed that for VI status evaluated by H&E, poor tumor differentiation (*P* = 0.001), perineural invasion (*P* = 0.021), and presence of lymph node metastasis (*P* = 0.038) were independent prognostic factors of worse disease-free survival. However, for VI status detected by H&E staining, these factors showed no statistical significance (*P* = 0.088).

In contrast, multivariate analysis of VI evaluated by dual CD31‒desmin immunolabeling showed that poor tumor differentiation (*P* < 0.001), perineural invasion (*P* = 0.019), and VI detected by dual immunolabeling (*P* = 0.009) were independent prognostic factors of worse disease-free survival.

## Discussion

VI was hypothesized to be the reason that pancreatic cancers have an aggressive behavior [11]. Detection of VI in pancreatobiliary tract cancers has been negatively associated with patient survival, and guidelines recommend that VI should be recorded in surgical pathology reports. H&E staining has been employed as the gold standard for assessing VI. However, this method is both labor-intensive and time-consuming to pathologists. In this study, we evaluated VI patterns and observed significant findings to help detect the patterns. The main findings of the present study are that both conventional and IN-like patterns are much more frequent than the destructive pattern assessed by H&E staining. Second, detection of VI is easier with single CD31 or dual CD31‒desmin immunolabeling than with H&E staining. Third, the destructive pattern of VI could be detected by H&E when the residual destructed smooth muscle layer of venous wall remained and was more easily detected with dual CD31‒desmin immunolabeling. Last, the presence of VI detected by dual CD31‒desmin immunolabeling was an independent worse prognostic indicator of patients with surgically resected pancreatobiliary tract cancers.

The prevalence of VI observed with conventional H&E staining of the validation set in the present study was 67% (40/61 cases), which is similar to that observed in previous reports that examined conventional H&E-stained slides [6]. In addition, we recently evaluated 319 pancreas cancer cases with a focus on various histologic features of VI evaluated using H&E-stained slides alone; we observed VI in 68% cases, and increased foci number of VI (≥3 foci) was associated worse clinical behavior and prognosis in patients with surgically resected pancreatic cancers [12]. With additional dual CD31‒desmin immunolabeling, we found more foci of VI than by H&E staining in the present study and dual CD31-desmin immunolabeling could more stratify for patients with surgically resected pancreatic cancers.

Endothelial markers, including CD31 and CD34, have been used for confirmation of VI in cancers of other organs, including colorectal, oral cavity, and breast cancers [13–15]. In the present study, we observed that CD31 immunolabeling could better detect the conventional pattern of VI than H&E staining. However, CD31 labeling was not helpful for identifying the IN-like and destructive patterns because of partial or total loss of CD31 expression on the endothelial side of the venous wall. This loss of CD31 expression of endothelial labeling in cancer invasion foci was previously described in lymphatics of papillary thyroid carcinoma and was explained as endothelial cancerization [16]. Similarly, we also observed that CD31 expression was lost at the points at which the cancer-cell clusters attached to the intimal layer of blood vessels but remained intact in areas where endothelial cells were not attached to cancer cells. Foci with the IN-like pattern of VI showed no CD31 immunolabeling because cancer cells completely covered the entire circumference of the endothelial portion of blood vessels. In this situation, additional single desmin labeling on consecutive serial sectioning could be helpful for detecting the IN-like pattern of VI. However, while desmin staining has a limitation that it is only useful for muscularized vessels, CD 31 staining has an advantage that it can confirm the capillary level of VI. Therefore, the best immunohistochemical method to detect VI could be dual CD31‒desmin immunolabeling, which was confirmed to detect more foci of VI, especially for the IN-like and destructive patterns.

The destructive pattern was observed in only 7.2% of the test set and 6.2% of VI of the validation set detected by H&E staining. However, detection of the destructive pattern of VI was increased up to 22.3% with single desmin immunolabeling in the test set and up to 29.8% with dual CD31‒desmin immunolabeling in the validation set. We categorized the destructive pattern as suspicious and definite destructive patterns: when the residual destructive smooth muscle layers of the venous wall were present on H&E slides, VI was categorized as the definite destructive pattern. In contrast, the suspicious destructive pattern was defined when identifying the definite residual destructive smooth muscle layers of vein was difficult on H&E-stained slides, so further single desmin or dual CD31‒desmin immunolabeling was performed to identify residual smooth muscle layer to confirm the destructive pattern as a definite destructive pattern. Among the 25 foci of suspicious destructive VI in the H&E-stained slides in the validation set, only 13 foci (52%) were confirmed as exhibiting the definite destructive pattern of VI dual CD31‒desmin immunolabeling. Instead, 36 additional foci exhibiting the destructive pattern were newly observed in the dual CD31‒desmin immunolabeled samples, which were not originally considered to be VI. These observations suggested that H&E staining has some limitations in the identification of the destructive pattern, and additional dual CD31‒desmin immunolabeling is helpful. The destructive pattern was characterized by luminal desmoplastic stromal reaction with tumor cells and inflammatory cell infiltrations with or without accompanying orphan artery/arteriole signs. These features were also observed close to areas of VI with a conventional or PanIN-like pattern [6], which suggests that one pattern could progress to another pattern. Recently, using *in vitro* organoid models, Nguyen et al. reported that pancreatic ductal adenocarcinoma cells invade the matrix and enter the lumina of vessels, where they ablate the endothelial cells, leaving behind tumor-filled luminal structures [17]. Their observations are consistent with our observation of destructive patterns in human pancreatobiliary tract cancers in human pathology samples. The destructive pattern of venous invasion may prohibit the appropriate delivery of chemotherapeutic agents via occlusion and the reduced venous route to pancreatobiliary tract cancers [11].

We found that the detection of VI using dual CD31‒desmin immunolabeling was more helpful for detecting destructive and IN-like patterns in the validation set. For example, the total number of foci of VI deficit for dual CD31‒desmin immunolabeling over H&E staining was 49 (58 vs. 9) for the destructive pattern, 12 (62 vs. 50) for the IN-like pattern, and -13 (74 vs. 87) for the conventional pattern in the validation set. Interestingly, additional dual CD31‒desmin immunolabeling was not helpful for identifying the conventional pattern of VI.

**Fig 9:**
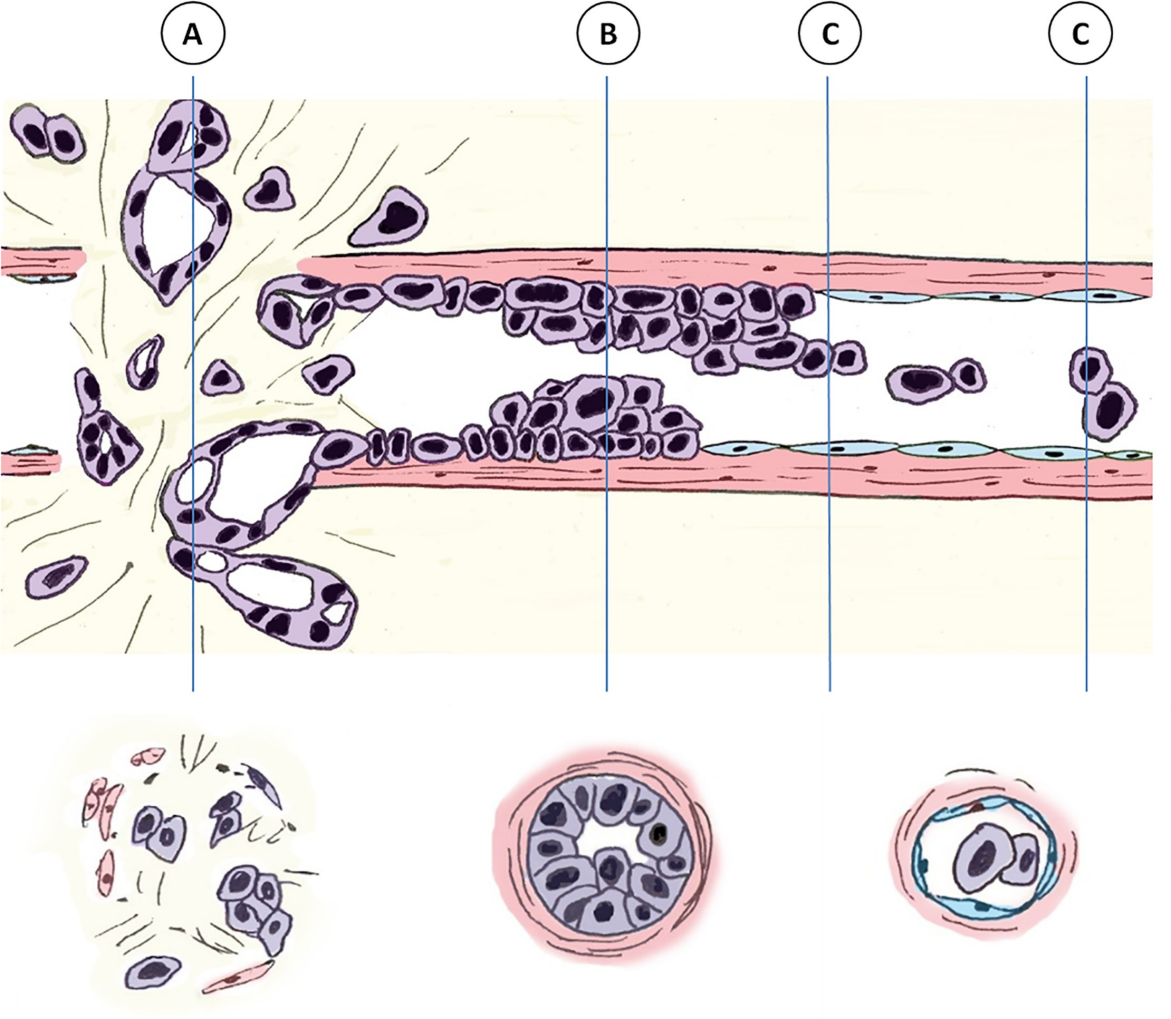
Diagram showing the proposed sequence of VI of pancreatobiliary tract cancers.

In the present study, we observed the presence of a mixed pattern of VI—destructive, IN-like, and conventional patterns. Furthermore, the destructive pattern of VI disappeared after several additional consecutive cut sections. In addition, by 3-dimensional (3D) reconstruction of the cleared pancreatic cancer tissue, we previously observed that neoplastic cells crossed the media of the involved veins as cords and tube-like growths of cancer cells (which can be translated as destructive pattern in the present study) rather than individual cells crossing the media of veins [18]. Cancer cells formed tube-like structures (which can be interpreted as IN-like patterns) and were found to grow within the venous wall. Single or clustered cancer cells were detached at the end of the tube-like growth of cancer cells (which can be interpreted as conventional pattern in the present study) [18]. When E-cadherin immunolabeling was performed, the neoplastic cells crossed the media of veins and formed tube-like structures within the venous wall with intact E-cadherin labeling in the large cohesive cluster of neoplastic cells, and loss of E-cadherin was observed only in the tips of small tongues of cells extending from these clusters [18]. Combining our present and previous observations, we hypothesized that these patterns may not be independent but may be related, and our hypothesized sequence of venous invasion patterns is depicted in **Fig 9**.

Recent 3D imaging of VI of pancreas cancers revealed that neoplastic cells that covered the vascular endothelial spaces extended for a distance with a mean length of approximately 560 μm [18]. The length of conventional pattern might be short. When we cut serial sections from H&E-stained slides contained conventional pattern of VI for making immunolabeling slides from FFPE tissue blocks, we observed that many foci of conventional pattern of VI were lost on dual CD31‒desmin immunolabeling slides in the present study. This result could be plausibly explained by the length of conventional pattern, which contained floating single or clustered cancer cells, are too short to left on the serial sectioned immunolabeling slides. In contrast, most of the foci of IN-like pattern were remained after serial sectioning and observed on dual CD31‒desmin immunolabeling slides. Therefore, most of the neoplastic cells within venous space is believed to form tubular space in 3D, which can be observed as IN-like pattern in 2D, to travel long distance for distance metastasis.

Not only presence of VI but also increased number of foci of VI are associated with worse survival in patients with pancreas cancers. Using H&E-stained slides alone, we observed that patients with pancreas cancer and ≥3 foci of VI had worse overall survival than those with 1–2 foci of VI or patients without VI in 319 surgically resected pancreatic cancer cases [12]. To identify the best cutoff point of the number of foci of VI based on dual CD31–desmin immunolabeling, further studies with large number of cases tested with dual CD31–desmin immunolabeling are required. Dual CD31–desmin immunolabeling may not be affordable for all pathology laboratories worldwide due to time and cost issues. However, if dual immunolabeling is not affordable, performing single desmin and CD31 labeling may be beneficial for patients with pancreatobiliary tract cancers because increased the number of foci of VI is known to be an important factor associated with worse survival in patients with surgically resected pancreatic cancers [12].

In summary, compared with H&E staining, more foci of VI could be detected with additional single CD31 or dual CD31‒desmin immunolabeling. The precise evaluation of VI with dual CD31‒desmin immunolabeling can provide additional prognostic information for patients with surgically resected pancreatobiliary tract cancers.

## Supporting information

S1 Data (XLSX)
